# Exosomal MIF Derived From Nasopharyngeal Carcinoma Promotes Metastasis by Repressing Ferroptosis of Macrophages

**DOI:** 10.3389/fcell.2021.791187

**Published:** 2021-12-31

**Authors:** Wenhui Chen, Fan Zuo, Kaiwen Zhang, Tian Xia, Wei Lei, Zixiang Zhang, Lili Bao, Yiwen You

**Affiliations:** ^1^ Department of Otorhinolaryngology Head and Neck Surgery, Affiliated Hospital of Nantong University, Nantong, China; ^2^ Institute of Otolaryngology Head and Neck Surgery, Affiliated Hospital of Nantong University, Nantong, China; ^3^ Department of Stomatology, Affiliated Hospital of Nantong University, Nantong, China

**Keywords:** NPC metastasis, exosome, MIF, macrophage, ferroptosis

## Abstract

Nasopharyngeal carcinoma (NPC) is the most common malignant tumor of the head and neck cancer (HNC). Metastasis is the main cause of treatment failure. However, the molecular mechanism for NPC metastasis is still unclear. As one of the most common host immune cells in the tumor microenvironment, macrophages have been proven to regulate metastasis. Besides, exosomes are the important bridge connecting various cells in TME. Currently, the role of NPC-exos on macrophages and their impact on metastasis remains to be unexplored. In this study, we found that MIF was highly expressed in NPC cells, and the exosomes secreted by NPC cells could be taken up by macrophages, thereby, inhibiting the ferroptosis of macrophages and then promoting the metastasis of NPC. Targeting MIF may be a potential treatment to reduce the rate of metastasis.

## Introduction

Nasopharyngeal carcinoma (NPC) is a common malignant neck and head tumor in southern China and Southeast Asia. Unique regional distribution and a tendency toward early metastasis are the characteristics that distinguish NPC from other HNC (Smalheiser, 2008). Metastasis is the main reason for treatment failure for NPC, and 60–85% of patients already have clinical metastasis at the time of diagnosis (Ivanovska and Cleary, 2008). With the continuous advancement of diagnosis and treatment, comprehensive treatment methods based on radiotherapy can often effectively control the primary tumor. Still, the treatment methods for metastatic tumors are limited, and the survival rate of NPC has not increased significantly for a long time (Kanellopoulou and Monticelli, 2008). Detecting early metastatic tendency and inhibiting the metastatic processes are still huge challenges for scientific research and clinicians.

Macrophage migration inhibitory factor (MIF) is an inflammatory cytokine discovered by Bloom and Bennett in 1966 that can inhibit the migration of macrophages. At the same time, MIF, as a pleiotropic chemokine, participates in many cellular processes, especially proliferation, differentiation, motility, matrix remodeling, metabolism, and micro vessel density of malignant lesions (Mitchell and Yaddanapudi, 2014). Early scholars believed that MIF was secreted by T cells, and it was a highly conserved protein in evolution (Mitchell and Yaddanapudi, 2014). Paralkar described the MIF gene in 1994 as follows: a small gene <1 kb located on chromosome 22, with a molecular weight of 12.5 kDa, containing 115 amino acids, and a trimer as the active form. MIF is secreted by many different types of cells and interacts with several receptors, which helps to explain the variety of biological functions. Receptors that interact with MIF include CD74, chemokine receptors CXCR2 and CXCR4 (Weiser et al., 1989; Bernhagen et al., 1993). MIF was bound to the extracellular domain of CD74, resulting in extracellular signal-regulated kinase (ERK) pathway activation (Leng et al., 2003). MIF-induced ERK activation through CD74 appears to depend on CD74, forming a complex with co-receptor CD44 (CD74/CD44) (Shi et al., 2006). In addition to ERK, stimulation of CD74 has been shown to trigger activation of the PI3K-Akt signal transduction cascade, NF-κB, and the AMP-activated protein kinase (AMPK) pathways. These pathways play essential roles in cell proliferation and survival (Su et al., 2017).

Subsequent studies have shown that this chemokine was secreted not only by lymphocytes but also by tumor cells (Costa-Silva et al., 2015), macrophages, epithelial cells, and endothelial cells (Conroy et al., 2010). The finding that MIF was released by pituitary cells and exerted the anti-inflammatory effects of corticosteroids, suggesting that MIF played a crucial role in inflammatory diseases (Bernhagen et al., 1993). Later, MIF was found in sepsis and autoimmune diseases and increased in the serum of patients with metabolic syndrome, and MIF was also involved in the formation of atherosclerosis (Zernecke et al., 2008; Kim et al., 2011). Increasing evidence showed that MIF was often overexpressed in cancer tissues and participated in the process of carcinogenesis. Kindt et al. found that the expression level of MIF in HNSC increased significantly (Kindt et al., 2013). At present, it is generally believed that MIF promotes tumor growth through various mechanisms, not only in cancer cells but also nearby and even distant cancer cells and noncancer cells.

Exosomes, small (30–150 nm) extracellular vesicles of endocytic origin, are present in all body fluids of cancer patients. Tumor cells produce large quantities of exosomes, which are called tumor-derived exosomes (TEX), and which are of special interest, as their molecular and genetic contents in part resemble those of the parent cell. Thus, TEX are considered to be similar to tumor cells from which they originate (Whiteside, 2018). Exosomes play a key role in cell-to-cell communication between cancer cells and their microenvironment, conveying information through their cargo, such as proteins, microRNAs, and messenger RNAs (Choi et al., 2013). Tumor cells secrete exosomes containing noncoding RNA and proteins with special functions, regulating tumor cell proliferation, apoptosis, invasion, and migration (Whiteside, 2018; Rodrigues et al., 2019). Senlin Zhao et al. found that exosomal miR-934 could promote colorectal cancer liver metastasis (CRLM) by regulating the crosstalk between colorectal cancer (CRC) cells and TAMs (Zhao et al., 2020). An EV-associated function, like a soluble cytokine-dependent function, should be observed between two cells that are not in direct contact with each other. Therefore, it should be obtained when the EV-donor and an EV-recipient cell are cultured *in vitro* at a distance, through transwell coculture systems or more sophisticated microfluidics-based culture devices, or by incubating the recipient cells with the medium conditioned by the donor cells (Théry et al., 2018). In our previous study, we succeeded in isolating, extracting, and identifying exosomes derived from serum or cell supernatant in NPC. And we found metastasis-associated miR-23a from NPC-derived exosomes could be delivered to HUVECS, mediating angiogenesis by repressing TSGA10. Besides, exosomal miR-17-5p promoted angiogenesis in NPC via targeting BAMBI (Bao et al., 2018; Duan et al., 2019). MIF acts as a macrophage migration inhibitor, Mariana et al. found that MIF could regulate the polarization of macrophages (Barbosa de Souza Rizzo et al., 2018). In addition, Deng Pan et al. confirmed that MIF was highly expressed in exosomes of non-small cell lung cancer (NSCLC) (Pan et al., 2019).

Ferroptosis is a process of programmed cell death that depends on iron and can react with excess oxygen (ROS) through the Fenton reaction (Latunde-Dada, 2017; Xu et al., 2019). Ferroptosis has nothing to do with apoptosis characteristics and is not inhibited by inhibitors that prevent necrosis or apoptosis; therefore, ferroptosis is different from other forms of cell death (Dixon et al., 2012a). The basis for the regulation of metabolism includes the production of iron-dependent (phosphorus) lipid hydrogen peroxide and the reduction of glutathione peroxidase 4 (GPX4) activity (Friedmann Angeli et al., 2014). The latter prevents the decay of hydroperoxyl lipids to oxidatively truncated reactive electrophilic species (Wenzel et al., 2017; Stoyanovsky et al., 2019). Chih-Chan Lee et al. and RUYI LI found that TAM could significantly promote metastasis in mice, and after depleting macrophages, compared with the control group, tumor metastasis in mice was significantly reduced (Lee et al., 2018; Li et al., 2019). All these indicate that the survival of macrophages deeply affects the progress of tumors and provides theoretical support for our study of ferroptosis and metastasis of macrophages.

In this study, we want to explore the expression and biological roles of MIF in NPC. And we will examine its effect on the ferroptosis of macrophages by exosomes to explain the underlying mechanism.

## Materials and Methods

### Cell Culture and Collection of Human NPC Specimens

The human NPC cell lines CNE-1 (highly differentiated), CNE-2 (poorly differentiated), 5–8F (high tumorigenesis and metastasis), 6–10B (low tumorigenesis and low metastasis) and the normal nasopharyngeal epithelial cell line NP69 were all gifts from Xiang-Ya and Sun Yat-sen University School of Medicine. NPC cells were cultured in RPMI 1640 (Biological Industries Israel Beit-Haemek, 01–100-1ACS) containing 10% FBS (Biological Industries Israel Beit-Haemek, 04–001-1ACS). NP69 cells were cultured in keratinocyte-SFM (Thermo Fisher Scientific, 17,005–042). THP-1 human monocytic leukemia cells were purchased from the American Type Culture Collection (ATCC) and cultured in RPMI 1640 medium containing 10% fetal bovine serum (FBS) and penicillin/streptomycin. Each cell line was maintained at 37°C in humidified air and 5% carbon dioxide according to the provider’s suggestions. All experiments were performed with mycoplasma-free cells.

Tissue and serum samples from pathologically confirmed cases of NPC were collected at the Affiliated Hospital of Nantong University following ethics committee approval (IRB number: 2018-L018). All included patients were informed and had not received any cancer therapies before biopsy. Detailed patient clinicopathological features are listed in Supplementary Table S1. The prognostic significance of MIF was assessed by Kaplan-Meier analysis. The X-tile Software (version 3.6.1; Rimm Lab; Yale School of Medicine) was used to define MIF low and high expression before analysis.

The study conformed to the standards set by the Declaration of Helsinki.

### Macrophage Differentiation

THP-1 cells were treated with 100 nM phorbol-12-myristate-13-acetate (PMA) for 1 day and then differentiated into M1 macrophages with LPS (50 ng/ml, Sigma) + rIFN-γ (20 ng/ml, PeproTech, Rocky Hill, United States). M2-polarized macrophages were induced by adding IL-4 (20 ng/ml, PeproTech) for 24 h.

### Transfection and Transduction With Lentiviral Vectors

CNE2 knockdown of MIF was obtained by infection with MIF shRNA lentiviral particles. The lentiviral vector and its corresponding control were obtained from Shanghai Genechem Co., Ltd. The specific vector used for cloning shRNA control and MIF shRNA: pRRLSIN-cPPT-U6-shRNA-SFFV-EGFP-SV40-puromycin. LV-MIF-RNAi (86638-12) CCC​GGA​CAG​GGT​CTA​CAT​CAA. The transfection efficiency was examined by fluorescence microscopy, qRT-PCR, and Western blotting.

### Processing and Culture of Macrophages

Macrophages were co-cultured with ISO-1(HY-16692,20uM, MCE) and human recombinant MIF factor (300–69,20 ng/ml, PeproTech) for 24 h. Ferrostatin-1 (Fer-1) and RSL3 were purchased from MCE.

### qRT-PCR Analysis

First, the cells were washed with precooled PBS three times to remove the serum. Then the remaining liquid was aspirated, TRIzol (Thermo Fisher Scientific, 15596018) was added, the cells were gently scraped off with a cell scraper, chloroform was added, followed by vortex and centrifugation to remove the top layer, and isopropanol was added followed by centrifugation and isolation of the precipitate, and reverse transcription was performed. The reverse transcription system was 20 μL, and qRT-PCR was performed after cDNA was obtained.

### BALB/c Nude Mice Animal Model

To establish the lung metastasis model, 2.0 × 10^6^ luciferase labeled CNE2 cells were injected via the tail vein of mice in 100 µL of PBS (10 per group). All mice were divided into three groups, each with 10 mice. Three days after the injection of cells, the first group was intraperitoneally injected with DMSO every 3 days, the second group was injected with PBS, and the third group was injected with ISO-1 (MedChemExpress, HY-16692). Then, IVIS Lumina Series III was used to measure the photon flux of the whole body of the mouse every week to determine whether the model was successful. To monitor the lung metastasis during the 6 weeks, fluorescence measurements were performed on each mouse weekly.

### Animal Ethics Statements

All animal experiments carried out in this project followed the NIH Guidelines with the ethical approval of the Administration Committee of Experimental Animals, Jiangsu Province, China (Approval ID: S20200323-089). *In vivo* studies were approved by the committee on the Ethics of Animal Experiments of Nantong University (RDD number: 20180227-008).

### Western Blotting Analysis

After the cells were lysed on ice for 30 min and centrifuged to collect the supernatant, the concentration was measured with the BCA protein assay (Thermo Scientific Pierce™ BCA protein assay, Waltham, MA), 5× sample buffer was added, and the cells were boiled at 100°C for 10 min. Samples were loaded on a 12% SDS-PAGE gel, and then the proteins were separated in running buffer. The protein sample was transferred from the gel to a PVDF membrane. The transmission system was used at 300 mA on ice for 1 h. The membrane was blocked with 5% skimmed milk at room temperature. The membranes were incubated with the specific primary antibody overnight at 4°C. The next day, membranes were washed with TBST and then incubated with the secondary antibody in 1 × TBS for 1 h at room temperature. Then, the membranes were rewashed in TBST and incubated with ECL from Millipore (Billerica, MA). Finally, the films were exposed to radiographic film in a dark room. Use of antibodies for western blotting: Anti-MIF antibody (ab7207), ABCAM, the dilution concentration is 1/500; Anti-Glutathione Peroxidase 4 antibody (ab231174), ABCAM, the dilution concentration is 1/500; Beta Actin Monoclonal Antibody, CloneNo:2D4H5,Proteintech, the dilution concentration is 1/600. Anti-Flotillin 1 antibody (EPR6041) (ab133497), the dilution concentration is 1/200. Anti-CD9 antibody (EPR23105-121) (ab236630), ABCAM, the dilution concentration is 1/500; Anti-TSG101 antibody [EPR7130(B)] (ab125011), ABCAM, the dilution concentration is 1/500.

### Immunofluorescence

After the cells were prepared on the plate, they were fixed with 4% paraformaldehyde, blocked, and incubated with anti-MIF antibody (ab7207, ABCAM, 1/200) overnight. The sections were stained with anti-rabbit or anti-mouse Alexa Fluor 488 IgG (Proteintech), counterstained with DAPI, washed 3 times with PBS, mounted with an anti-fluorescence attenuation mounting plate, and observed with a fluorescence microscope.

### Immunohistochemistry

Standard immunohistochemistry (IHC) methods were used to analyze human nasopharyngeal carcinoma specimens and lung metastasis specimens. Briefly, 5 μm thick sections were deparaffinized and then hydrated, and endogenous peroxidase activity was blocked with 3% hydrogen peroxide. The antigen recovery process was carried out in boiling water, blocked by peroxidase for 10 min, and then blocked with 10% BSA blocking buffer. The sections were incubated with anti-MIF (ab7207, ABCAM, 1/200), anti-INOS (ab115819, ABCAM, 1/100), anti-F4/80 (ab111101, ABCAM, 1/100) and anti-GPX4 (ab231174, ABCAM, 1/100), followed by secondary antibody and DAB. Then, hematoxylin was used to stain the nuclei, rinsed with running water for 40 min, finally, the gradient was reversed and observed under a microscope. Stained slides were assessed according to the staining intensity (extremely strong: four; strong: three; moderate: two; and weak: one) and the abundance of positive cells (≤5%: 0; 6–25%:1; 26–50%: 2; 51–75%:3, and ≥76%: 4) by two pathologists blind to the patient’s clinicopathological information. Scores of 0–8 were defined as low expression, and 9–16 were defined as high expression.

### Transmission Electron Microscopy

The isolated exosomes were mixed with 4% paraformaldehyde. Then, the exosomes were dropped onto an electron microscope grid coated with formvar-carbon and fixed with 1% glutaraldehyde for 10 min. The samples were negatively stained with 2% uranyl acetate solution. Images were acquired by a Tecnai transmission electron microscope at 163 kV.

### Exosome Isolation, Labeling

The detailed protocol of continuous ultracentrifugation for extracting exosomes can be shown in our previous work (Bao et al., 2018). The exosomes were labeled with a PKH-67 labeling kit (Sigma-Aldrich) to observe the uptake of exosomes. After CNE2 cells were cocultured with the labeled exosomes for 1 h, CNE2 cells were fixed, and the nuclei were stained with DAPI. Finally, images were collected with a Leica TCS-SP5 LSM microscope. In the subsequent uptake experiment of co-cultured exosomes, we added 80 ug of exosomes per 1 × 10^6 cells.

### Detection of Intracellular Fe^2+^ Using FerroOrange

Macrophages were seeded on a 96-well plate in a 37°C incubator. The cells were washed with the serum-free medium three times. Then, the serum-free medium was added to the cells. Ammonium iron (II) sulfate prepared with ddH_2_O (10 mmol/L) was added to the cells (final concentration: 100 μmol/L). The cells were incubated for 30 min and washed with HBSS three times. FerroOrange (1 μmol/L) was added to the cells as HBSS solution, and then the cells were incubated for 30 min in a 37°C incubator equilibrated with 95% air and 5% CO_2_. The cells were observed under a confocal fluorescence microscope.

### Intracellular ROS Detection

Intracellular ROS production in macrophages was measured by a dihydroethidium probe kit (DHE, BestBio, Beijing, China). Adherent macrophages were digested with trypsin and resuspended in 500 µL of prechilled PBS, probes were added and incubated for 1 h at 37°C in the dark, and 480–535 nm wavelength excitation was used to detect cellular ROS content.

### CCK8 Assay

First, we seeded 10,000 macrophage cells on a 96-well plate, then added different drugs and incubated them in a cell culture incubator (37°C, 5% CO2). After 18 h, aspirated the medium and washed it with PBS. Then Added 1/10 volume of CCK8 solution to the medium without serum, incubated for 2 h in the dark, and then detected its absorbance at 450 nm in a microplate reader, and recorded the value.

### Transwell Assay

Migration assay was performed using Transwell inserts (Corning, 3422) with a pore size of 8 µm. A total of 5 × 10^4^ cells suspended in medium without serum were added in the upper chambers, and the medium containing 10% fetal bovine serum was added to the lower chambers. After 16 h of incubation, the cells attached to the upper side were removed, and the cells attached to the underside of the membrane were fixed and stained with crystal violet. The invasion experiment is based on the migration experiment. Matrigel (Corning, 356234) was spread before the cells were seeded in the upper chamber. Digital images were obtained from the membranes, and five random fields were counted per chamber.

### Statistical Analysis

Statistical analysis was performed with GraphPad Prism 8 software. Data are presented as the mean ± SD. The significance of mean values between the two groups was analyzed using Student’s *t*-test. The differences between individual groups were analyzed by one- or two-way ANOVA. The differences were considered significant when the *p* value was less than 0.05.

## Results

### MIF Expression in Human Nasopharyngeal Carcinoma Is Higher Than That in Normal Cells

**FIGURE 1:**
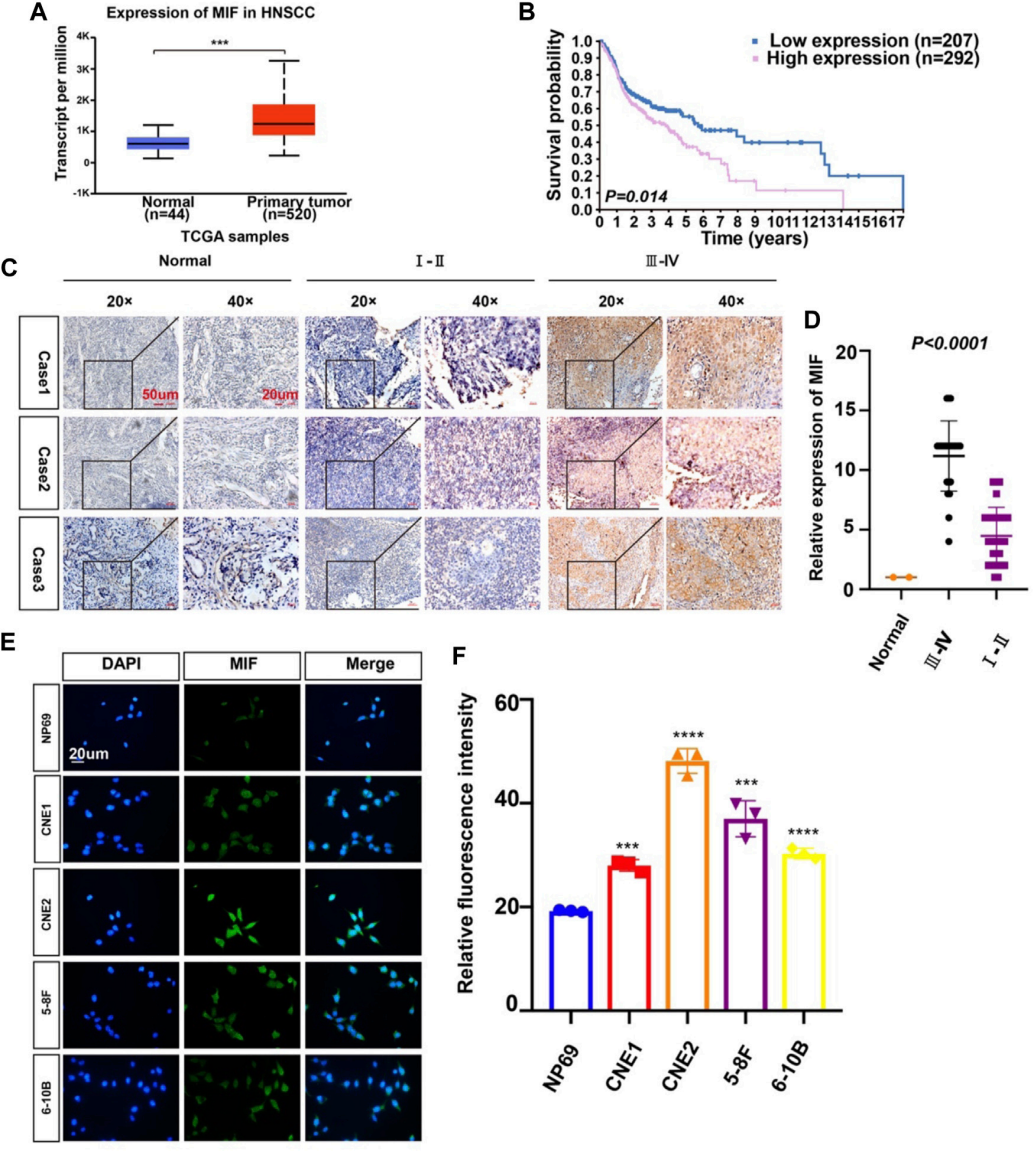
MIF expression in human nasopharyngeal carcinoma is higher than that in normal tissues. **(A)** Statistical comparison of MIF expression across TCGA database, one-way ANOVA. **(B)** Statistical analyses of the association of MIF expression with the survival time of the patients. Log-rank test. **(C)** Immunohistochemical staining of MIF in patient specimens of different stages. **(D)** The IHC staining score of MIF in NPC tissues was defined as low expression (scores of 0–8) or high expression (scores of 9–16) by X-tile Software. Then, Kaplan-Meier analysis was used to compare MIF IHC staining scores of patients in different stages. e MIF levels in NP-69 and NPC cell lines were examined by IF (unpaired t-test). CNE-1, CNE-2, 5–8F, and 6–10B are human NPC cell lines; NP-69 is an immortalized normal nasopharyngeal epithelial cell line. Scale bar:20 um. f Statistics of fluorescence intensity through ImageJ, unpaired t-test. All graphs show the mean ± SEM of at least three independent experiments. *p < 0.05, **p < 0.01, ***p < 0.001.

To identify possible genes related to NPC, we extracted data on HNC from TCGA. The level of MIF in HNC is higher than normal controls (Figure 1A), patients with higher MIF levels had a poor clinical prognosis relative to patients with lower MIF levels in HNSCC (Figure 1B), and MIF can be an important biomarker in various malignant tumors such as breast cancer, pancreatic ductal adenocarcinoma, and bladder cancer. We chose MIF for further research. Then, to further explore the level of MIF in NPC, immunohistochemistry (IHC) was performed. The results showed higher expression of MIF in NPC patients vs. normal controls and higher expression in clinical stage III-IV NPC patients than in clinical stage I-II patients (Figures 1C,D), which suggested that the upregulation of MIF level may be closely related to end-stage nasopharyngeal carcinoma. The expression level of MIF was further verified by immunofluorescence (IF), showing that MIF was highly expressed in NPC cell lines (Figures 1E,F). In addition, we found that CNE2 cells had the highest MIF level in NPC cell lines, so CNE2 cells were used in subsequent experiments.

### Overexpression of MIF Is More Prominent in NPC Patients With Metastasis

**FIGURE 2:**
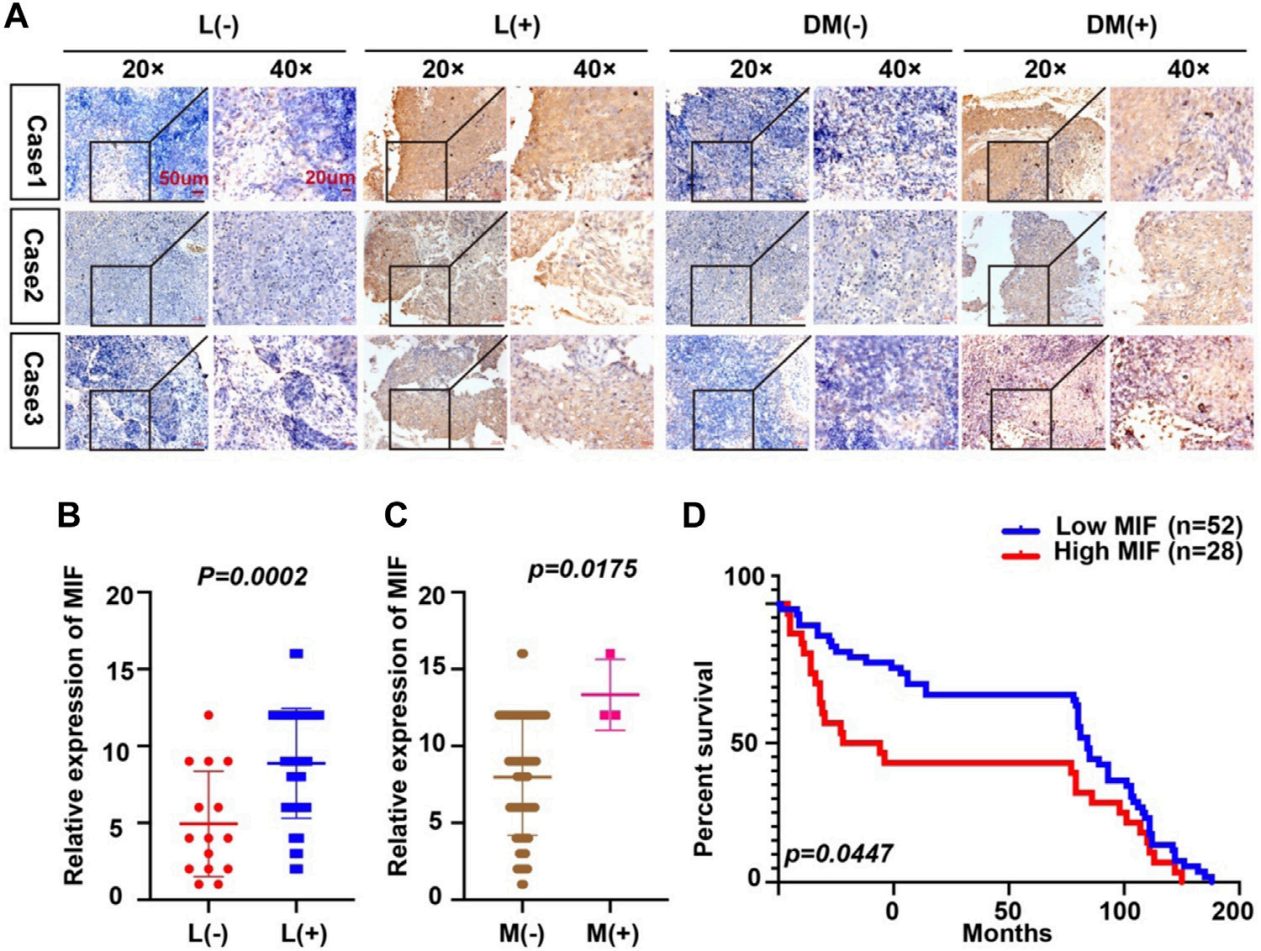
Overexpression of MIF is more prominent in NPC patients with metastasis. **(A)** Representative IHC images of MIF staining in tissues collected without lymph node metastasis, with lymph node metastasis, without distant metastasis, and with distant metastasis specimens from NPC patients. **(B–D)** Statistical analyses of the association of MIF expression with lymph node metastasis, distant metastasis and prognosis and Kaplan-Meier analysis. All graphs show the mean ± SEM of at least three independent experiments. *p < 0.05, **p < 0.01, ***p < 0.001.

Because the expression of MIF was higher in terminal patients, we speculated that MIF expression might be related to metastasis. To verify this conjecture, we compared the MIF staining intensity of patients with metastasis and patients without metastasis. We found that the MIF expression of patients with lymphatic metastasis was significantly higher than patients without lymphatic metastasis. Also, the MIF expression of patients with distant metastasis was notably higher than patients without distant metastasis (Figures 2A–C). In addition, the survival rate of patients was closely related to the expression of MIF. The higher the level of MIF expression was, the worse the prognosis of the patients (Figure 2D). The relevant clinical data were sorted in Table 1. Briefly, elevated MIF expression was significantly associated with tumor stage (*p* < 0.0001), lymph node metastasis (*p* = 0.0002), distant metastasis (*p* = 0.0175) and the survival rate of patients (*p* = 0.0447) but not with other clinicopathological parameters, including age and sex (Table 1).

**TABLE 1:** Relationship between MIF expression and clinical pathological factors of nasopharyngeal carcinoma.

Clinical indicators	Total	Low scoring	High scoring	c^2^	P
Age					
≥55 years old	41	22	19	0.003197	0.6184
<55 years old	39	16	23	—	—
Gender					
Male	56	29	27	0.004797	0.5415
Female	24	9	15	—	—
Stage					
Ⅰ-Ⅱ	51	32	19	0.2502	<0.0001****
Ⅲ-IV	29	6	23	—	—
Lymph nodes					
L (−)	15	11	4	0.1642	0.0002***
L (+)	65	27	38	—	—
Prognosis					
Dead	36	12	24	0.1143	0.0022**
Alive	44	26	18	—	—
Metastasis					
M (+)	3	0	3	0.07019	0.0175*
M (−)	77	38	39	—	—

*p value below 0.05 was considered statistically significant.

### MIF Promotes Lung Metastasis of NPC

**FIGURE 3:**
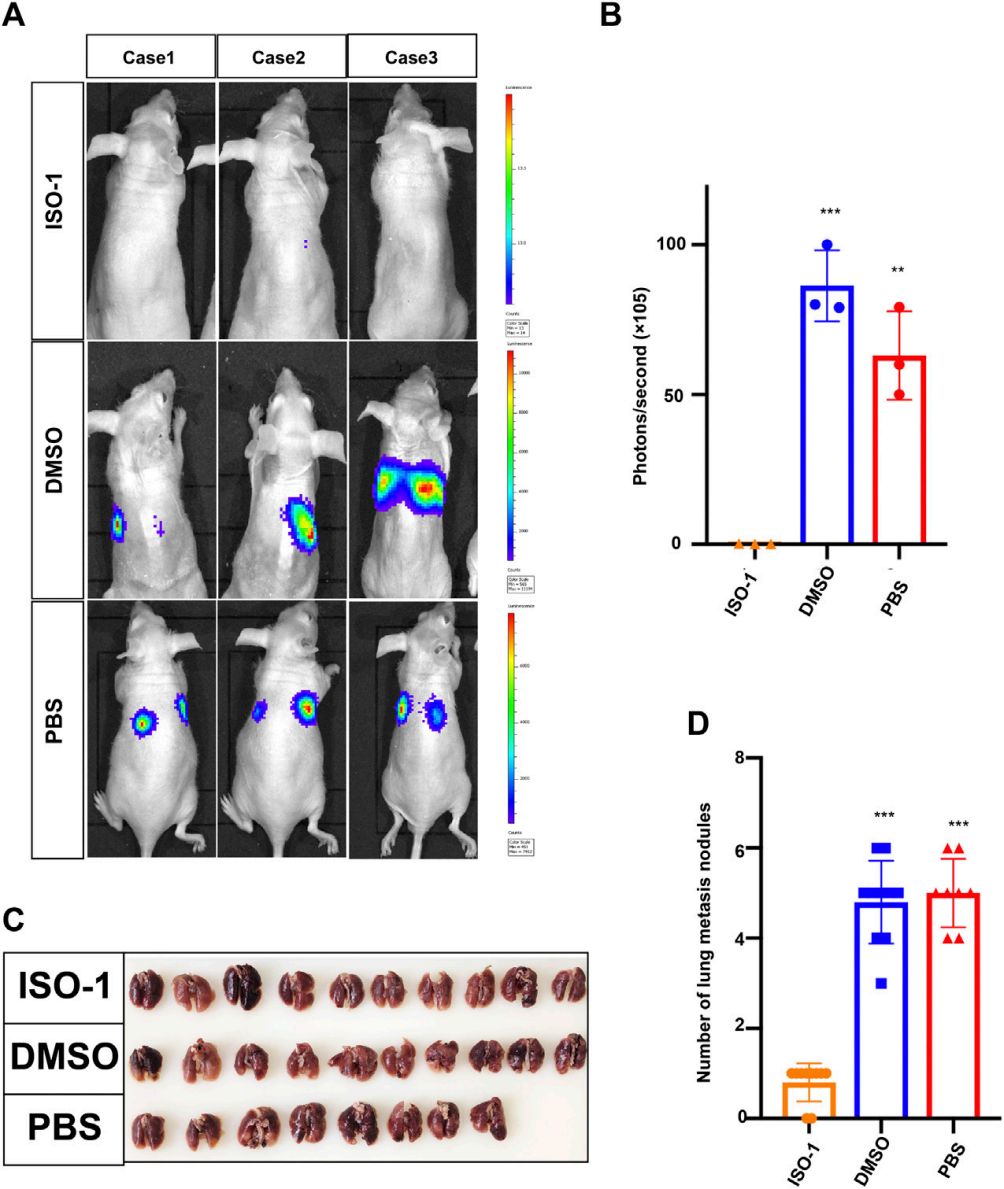
MIF promotes lung metastasis of NPC. **(A)** Visualization of lung metastasis after intravenous injection of CNE2 cells and then separate injections of ISO-1, DMSO, and PBS into BALB/c mice. Representative images of bioluminescence signals and normalized photon flux are shown. **(B)** Graph representing the mean intensity of fluorescence 6 weeks after tumor injection (**p < 0.01, ***p < 0.001, t-test). **(C)** Representative images of lung metastasis in nude mice that resulted from the injection of ISO-1, DMSO, and PBS after intravenous injection of CNE2 cells. **(D)** Statistical analysis of the number of lung nodules in the ISO-1, DMSO, and PBS groups (***p < 0.001, t-test).

Since the upregulation of MIF was significantly related to advanced disease stage, metastasis, and poor survival rate, we used a BALB/c mouse animal model to determine the function of MIF *in vivo*. Firstly, we injected luciferase labeled CNE2 cells into nude mice through the tail vein. Three days after the injection, the first group was intraperitoneally injected with DMSO every 3 days, the second group was injected with PBS, and the third group was injected with ISO-1 (an inhibitor of MIF). The growth of the tumor was then monitored by measuring the amount of bioluminescence (BLI). We found that the MIF inhibitor significantly inhibited the metastatic ability of CNE2 cells (Figures 3A,B). The number of lung metastasis nodules in this group of mice injected with the MIF inhibitor ISO-1 was significantly less than the other two groups (Figures 3C,D). This shows that MIF is a high-risk factor that promotes the metastasis of nasopharyngeal carcinoma.

### MIF Inhibits Ferroptosis of Macrophages *in vitro*

MIF is also known as an inhibitory factor of macrophage migration and has been confirmed to be related to the migration function of macrophages. Then, we speculated that MIF was very likely to regulate the function of macrophages, thus affecting the metastasis of NPC. Stoppe C et al. found that MIF might be involved as a counter-regulatory factor to regulate tubular necroptosis or ferroptosis (Averdunk et al., 2020). This provided clues for us to explore the relationship between MIF and ferroptosis.

**FIGURE 4:**
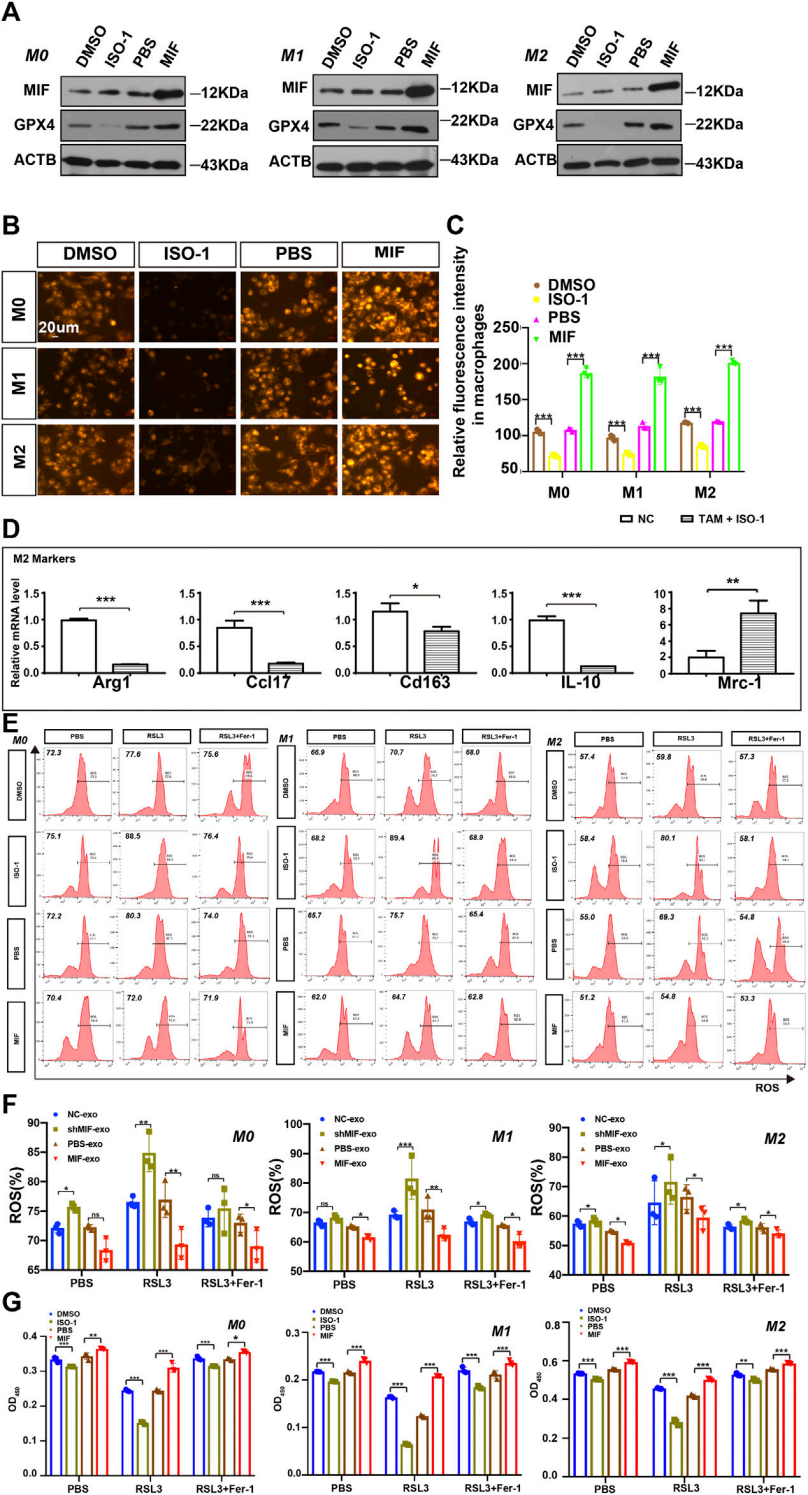
MIF inhibits ferroptosis of macrophages *in vitro*. **(A)** WB analysis of the relationship between GPX4 and MIF expression levels in M0, M1 and M2 macrophages. **(B)** FerroOrange experiment involving staining of divalent iron in macrophages. Scale bar:20 um. **(C)** Statistical analysis of the fluorescence intensity of ferrous iron. **(D)** After ISO-1 was added, macrophage polarization was detected by qRT-PCR. **(E,F)** Flow cytometry of ROS in macrophages (Two-way ANOVA). **(G)** Cell death was estimated by CCK8 assay (Two-way ANOVA). All graphs show the mean ± SEM of at least three independent experiments. *p < 0.05, **p < 0.01, ***p < 0.001.

Tumor-associated macrophages (TAMs) are one of the most common host immune cells in the tumor microenvironment and can accelerate tumor growth, remodel the extracellular matrix, and promote cancer metastasis. Since macrophages are the most abundant iron storage cells in the human body, we speculated that MIF might regulate the ferroptosis of macrophages. We used a functional inhibitor of MIF (ISO-1) and MIF recombinant factors to treat macrophages, and the results showed that in M0, M1, or M2 macrophages, the expression of GPX4 decreased with the inhibition of MIF function. Still, the recombinant factor MIF restored the expression of GPX4 (Figure 4A). As an important part of the ferroptosis loop, ferrous iron plays the same important role as GPX4. In the experiment measuring divalent iron, after inhibiting the function of MIF, the content of divalent iron in macrophages decreased, while the recombinant factor MIF restored this effect (Figures 4B,C). Alexandr A. Kapralov et al. found that M1 polarized macrophages were the most resistant to RSL3-induced ferroptosis than M0 or M2 cells (Kapralov et al., 2020). Therefore, we were curious about the relationship between the ferroptosis and polarization of macrophages. We cocultured M0 macrophages with ISO-1 and found that after ISO-1 was added, the level of M0 macrophage differentiation into the M2 type decreased (Figure 4D). It suggested that MIF might promote the M2-type polarization of macrophages. Next, we endeavored to test the sensitivity of macrophages to a standard pro-ferroptosis treatment with a specific GPX4 inhibitor, RSL3 (1*S*,3*R*)-2-(2-chloroacetyl)-2,3,4,9-tetra-hydro-1-(methoxycarbonyl) phenyl-1*H*-pyrido (3,4-b) indole-3-carboxylic acid). We measured the ROS content of each group of macrophages by flow cytometry and we found that after inhibiting the MIF function of macrophages, the intracellular ROS content was significantly increased, and the recombinant factor MIF greatly inhibited ROS (Figures 4E,F). However, the increase in ROS caused by RSL3 could be effectively prevented by a selective ferroptosis inhibitor, ferrostatin-1 (Fer-1). The CCK8 experiment revealed that the ISO-1 treated cells were vulnerable and consistently responded with a loss of viability, whereas the MIF treated cells exerted high resistance to ferroptosis (Figure 4G). Above results proved that MIF could inhibit ferroptosis in macrophages. At the same time, we found that in the absence of RSL3, high MIF levels also decreased ROS content or cell death in macrophages. According to the decreasing or increasing rate, we suggested that MIF mainly regulated ferroptosis nor other forms of cell death.

### MIF can Be Transferred From NPC Cells to Møs *via* Exosomes

**FIGURE 5:**
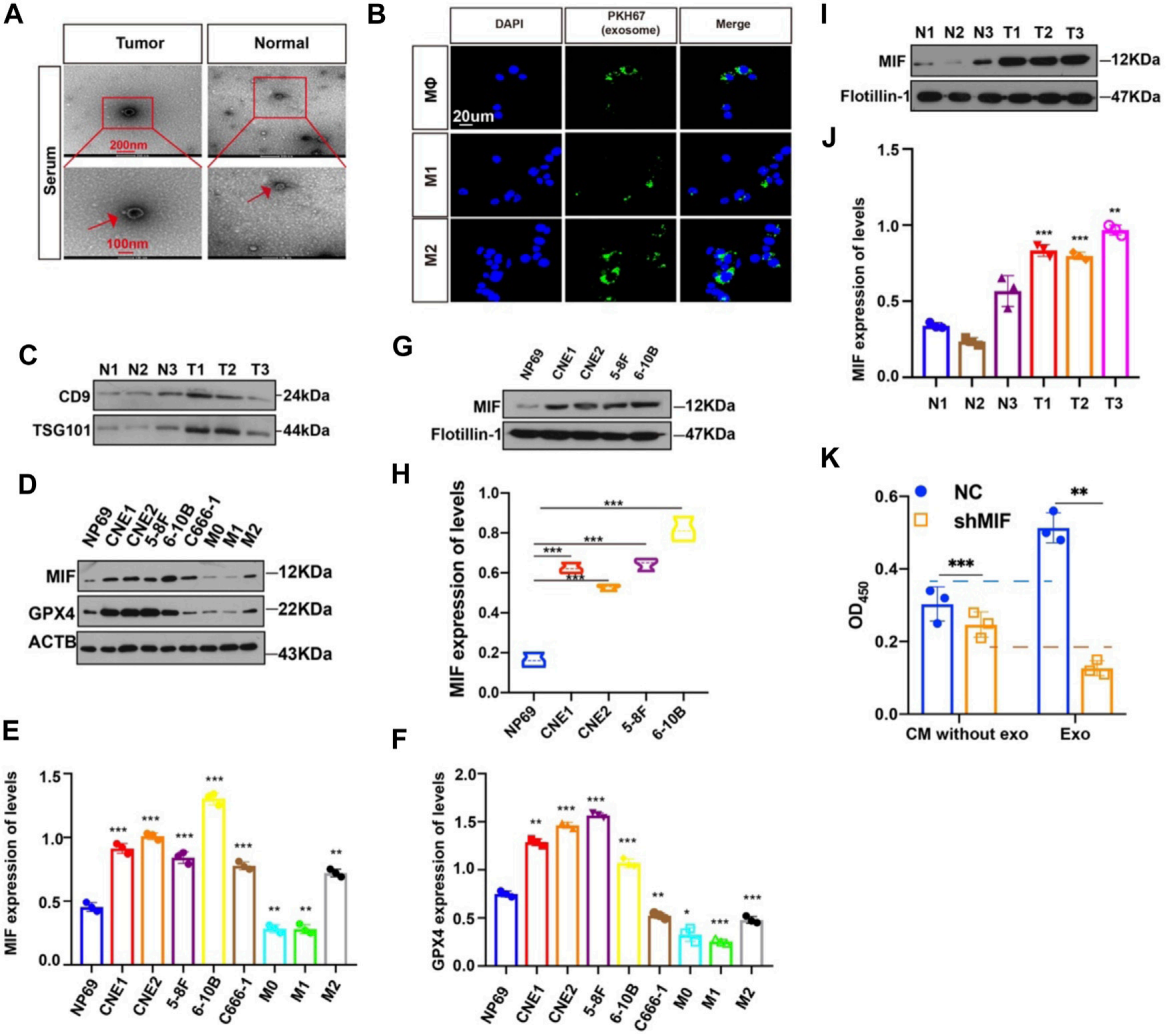
MIF can be transferred from NPC cells to Møs *via* exosomes. **(A)** Representative electron microscopy images of NPC-exos and Normal-exos. **(B)** Uptake of exosomes in macrophages by confocal microscopy. Exosomes are derived from the serum of NPC patients. Blue: DAPI staining; green: PKH67-labeled exosomes. Scale bar:20 um. **(C)** Validation of serum-derived exosomes markers **(D–F)** WB analyses of MIF and GPX4 expression in NPC cell lines, NP69 cells and macrophages (*p < 0.05, **p < 0.01, ***p < 0.001, t-test). **(G,H)** Analysis of the expression level of MIF in exosomes of NPC and normal cells (t test). **(I,J)** Analysis of the expression level of MIF in exosomes in serum from three cases of nasopharyngeal carcinoma and normal controls (t test). **(K)** CM without exo: the supernatant derived from the CNE2 control and transfected knockdown MIF lentivirus, and the exosomes were removed. exo: Exosomes. All graphs show the mean ± SEM of at least three independent experiments.

Then, how did highly expressed MIF in NPC reach macrophages? We had previously successfully extracted and identified exosomes derived from NPC and found that exosomes in the microenvironment of NPC played a significant role in cell-to-cell communication, thus promoting angiogenesis, invasion, and metastasis (Bao et al., 2018; Shan et al., 2018; Duan et al., 2019; Zhang et al., 2021). To determine whether exosomes mediate the transfer of MIF to macrophage, we isolated exosomes from the serum of normal people and NPC patients. The characteristic features of exosome lipid bilayer membranes can be identified by transmission electron microscopy (TEM) (Figure 5A). In addition, after M0, M1, and M2 macrophages were cocultured with PKH67-labeled exosomes for 2 h, the uptake of PKH67-labeled exosomes was observed by fluorescence microscopy (Figure 5B). This indicated that exosomes derived from NPC could indeed be taken up by macrophages. We have also verified the serum-derived exosomes markers (Figure 5C). Next, we quantified the expression of MIF in normal nasal mucosal cells, NPC cell lines, and macrophages. We found that the MIF level of NPC cell lines was significantly higher than that of the normal nasal mucosal cell line NP69 and M0, M1, and M2 macrophages (Figures 5D,E), and GPX4 (glutathione peroxidase 4) expression was positively correlated with MIF (Figures 5D,F). The expression level of MIF in the exosomes of NPC cell lines was also significantly higher than normal (Figures 5G,H). To quantify the specific level of MIF in exosomes, the Western blotting results proved that the MIF level in patients with NPC was higher than that in healthy volunteers (Figures 5I,J). We also conducted the CCK8 experiment to compare the effects of soluble MIF and exosomal MIF on the viability of macrophages (under RSL3 treatment). Results showed that soluble MIF in the supernatant could also inhibit ferroptosis, but their influence on ferroptosis effectiveness was weaker than exosomal MIF (Figure 5K). This indicates that MIF derived from NPC may be delivered to macrophages through exosomes to play a role in regulating ferroptosis.

### Exosomal MIF Inhibits Ferroptosis of Macrophages *in vitro*

**FIGURE 6:**
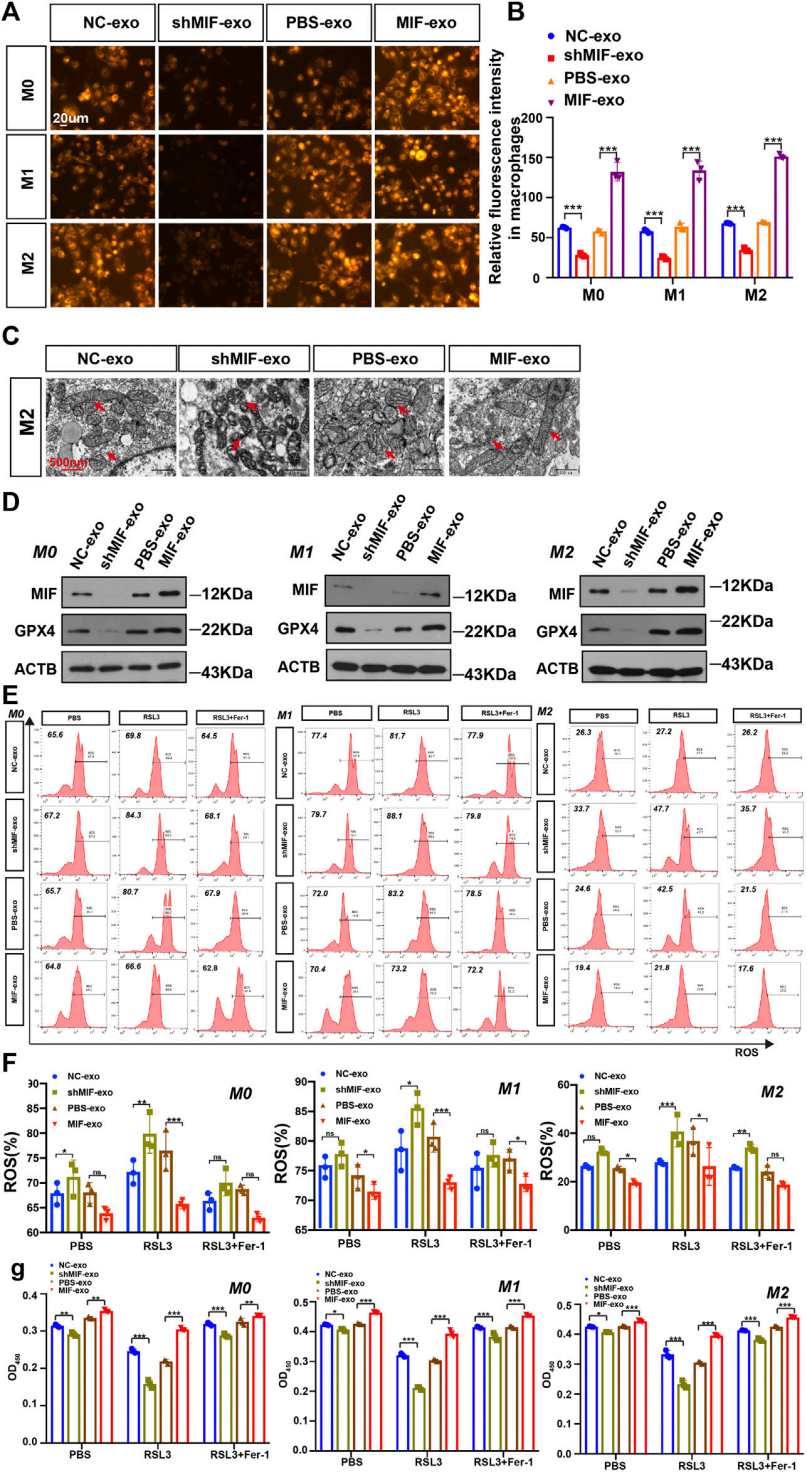
Exosomal MIF inhibits ferroptosis of macrophages *in vitro*. **(A)** FerroOrange experiment involving staining of divalent iron in macrophages. Scale bar:20 um. **(B)** Statistical analysis of the fluorescence intensity of ferrous iron. **(C)** Representative transmission electron microscopy (TEM) images of M2 macrophages. Scale bar:500 nm. Red arrows indicate mitochondria. **(D)** WB analysis of the relationship between GPX4 and MIF expression levels in M0, M1 and M2 macrophages. **(E,F)** Flow cytometry of ROS in macrophages (Two-way ANOVA). **(G)** Cell death was estimated by CCK8 assay (Two-way ANOVA). All graphs show the mean ± SEM of at least three independent experiments. *p < 0.05, **p < 0.01, ***p < 0.001.

To confirm the possible regulatory effect of MIF in exosomes on the ferroptosis of macrophages. We collected exosomes secreted by CNE2 cells transfected with knockdown MIF lentivirus and CNE2 cells cocultured with recombinant MIF. The results showed that in M0, M1, and M2 cells, the exosomes derived from the MIF knockdown group significantly reduced the content of divalent iron in the macrophages, and adding exosomes derived from the recombinant MIF group reversed this result, indicating that exosomal MIF can inhibit ferroptosis of macrophages (Figures 6A,B). It was also observed by transmission electron microscopy that after coculture with CNE2-derived exosomes and MIF knockdown, the mitochondria of macrophages decreased, the membrane density increased, and the outer mitochondrial membrane was broken. After adding CNE2-derived exosomes that were cocultured with recombinant factor MIF, the effect was reversed (Figure 6C). Similarly, the expression of GPX4 was the same as indicated above. After macrophages were cocultured with CNE2-derived exosomes that knocked down MIF, the expression of GPX4 decreased. However, after coculture with recombinant factor MIF, CNE2-derived exosomes increased GPX4 expression (Figure 6D). Next, we tested the ROS content in macrophages. Consistent with the above results, MIF in exosomes reduced the ROS content in macrophages (Figures 6E,F). Once again, the results of CCK8 also indicated that MIF strengthened the resistance of macrophages to ferroptosis (Figure 6G).

### MIF Inhibits Ferroptosis of Macrophages and Promotes Metastasis *in vivo*

**FIGURE 7:**
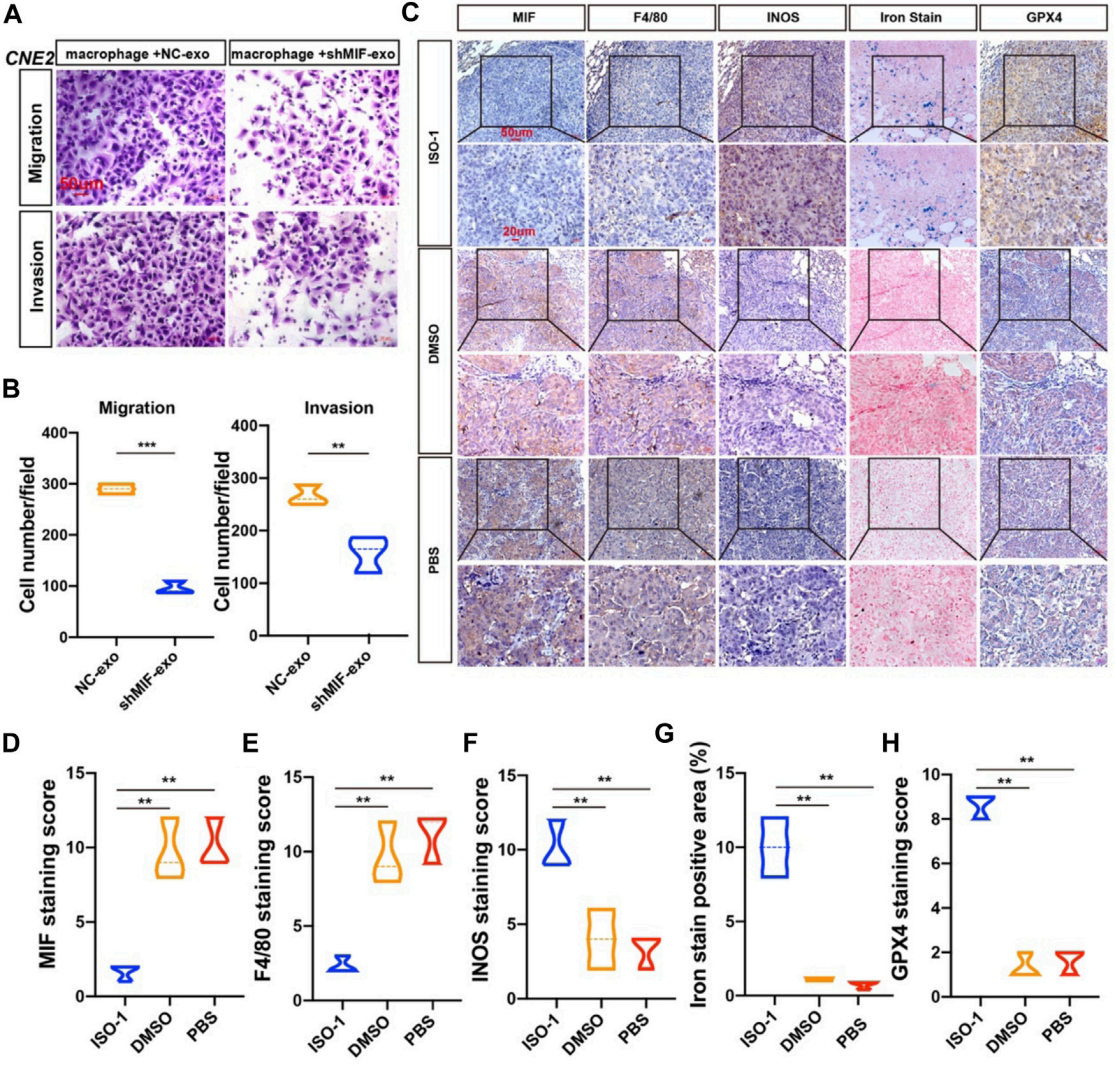
MIF inhibits ferroptosis of macrophages and promotes metastasis *in vivo*. **(A)** Collect the control group and the exosomes secreted by CNE2 transfected with knockdown MIF to co-culture with macrophages, collect the supernatant of the macrophages, and then perform the migration and invasion experiments of CNE2. Scale bar:50 um. **(B)** Quantification of migration and invasion (t test). All graphs show the mean ± SEM of at least three independent experiments. *p < 0.05, **p < 0.01, ***p < 0.001. **(C)** IHC analysis of the relationship between MIF and ferroptosis in the lungs of nude mice. **(D–H)** Quantification of IHC staining for MIF **(D)**, F4/80 **(E)**, INOS **(F)**, Iron stain **(G)** and GPX4 **(H)**, INOS: marker of M1 macrophages. F4/80: marker of total macrophages. Expression using one-way ANOVA (**p < 0.01).

To verify the effect of macrophages on the migration and invasion of CNE2 after ingesting the exosomes secreted by CNE2, we did a chamber assay. The results showed that macrophages exposed to exosomes with low MIF expression significantly reduced the migration and invasion ability of CNE2 (Figures 7A,B). To further verify the relationship between MIF, macrophage ferroptosis, and metastasis, we performed immunohistochemistry of lung metastases in mice from Figure 3. Immunohistochemistry and iron staining showed that the expression of MIF was positively correlated with the number of M2 macrophages but was opposite to the number of M1 macrophages. After inhibiting MIF function, ferric iron in tissue increased significantly, but the expression of GPX4 was the lowest (Figures 7C–H). This proved that MIF promoted tumor metastasis by inhibiting ferroptosis in macrophages.

## Discussion

NPC is the most common malignant tumor of the head and neck. The combination of radiotherapy and chemotherapy is the standard treatment for this disease, but current radiotherapy and chemotherapy have limited efficacy for many patients (Zou et al., 2019; Hong et al., 2020). For example, 30–40% of patients will develop distant metastases within 4 years, and the prognosis is poor, mainly because tumor cells have developed resistance to radiotherapy and chemotherapy. The pretumor niche is a system affected by the interaction between cells, which is closely related to the progression and distant metastasis of the tumor. Therefore, a comprehensive understanding of the molecular mechanisms that promote the progression and metastasis of NPC can help in the design of more effective and targeted treatment strategies. In this study, we confirmed that MIF was highly expressed in NPC, and patients with high MIF level was positively correlated with the poor prognosis.

MIF has been reported as an oncogene in many cancers. In cell culture and animal models of genitourinary cancer, inhibiting MIF activity can reduce the malignant biological behaviors of cancer, such as cell proliferation, angiogenesis, and tumor aggressiveness. MIF also regulates signaling pathways, such as extracellular signal-regulated kinase (ERK) protein-regulated signaling pathway kinase B and p53, to reverse immunosuppression. Many potential therapies to block the effects of MIF have been developed. Antibodies against MIF have been used in ongoing or recently completed phase I clinical trials. These cancer studies use anti-CD74-based antibody-drug conjugates to increase drug uptake or monoclonal antibodies against MIF or CD74 in cancers, including metastatic colon cancer, B-cell lymphoma, and other solid malignancies. Considering that MIF staining in tumors from patients could also be contributed by stromal/hematopoietic cells, it was plausible that the cooperation or synergism between tumor-secreted MIF and microenvironment MIF could fuel tumor progression and metastasis.

Cancer immunotherapy, which uses immune cells to fight tumors, has proven to be a powerful weapon against tumors and has been gradually used in clinical diagnosis and treatment (Lan et al., 2019). However, due to the lack of appropriate methods to target the tumor microenvironment, most patients with solid tumors have made little progress in this area. Local immune suppression can be reprogrammed to suppress the tumor environment, thereby restoring antitumor immunity. Macrophages are important cells in the body that deal with iron metabolism. They absorb, metabolize, store, and export iron to meet the needs of surrounding cells. The iron metabolism of macrophages can change their polarization balance. Macrophages decide whether to differentiate towards M1 or M2 according to different microenvironmental stimuli. In previous reviews, there were many studies on iron metabolism in tumor cells, but there are relatively few studies on the effects of ferroptosis in macrophages on tumor progression. M1-type macrophages exhibit strong iron absorption and storage capacity while showing a weakened iron release phenotype. A recent study indicated that when the iron was overloaded, the production of reactive oxygen species increased the differentiation of macrophages into a pro-inflammatory phenotype (Zhou et al., 2018). TAMs exhibit strong M2 polarization in most malignant tumors, which may be attributed to this “iron supply” feature that promotes cancer (Cairo et al., 2011). Bong-Sung Kim et al. found that the downregulation of MIF-2 in fat tissue potentially increased pro-inflammatory macrophage polarization (Kim et al., 2020). Gabriela F. de Souza et al. demonstrated that MIF was expressed during RSV infection and controlled the release of pro-inflammatory cytokines from macrophages in an *in vitro* model. MIF was essential for the release of TNF-α, MCP-1, and IL-10 triggered by RSV in macrophages (de Souza et al., 2019). Zhang Z et al. found that MIF inhibitor Z-590 possessed potent anti-arthritic activity through suppression of macrophage activation and could be a potential treatment for rheumatoid arthritis (RA) (Zhang et al., 2018). All these studies have shown that MIF was closely related to macrophages and affected various functions of macrophages, such as the polarization of macrophages and the release of cytokines. This provided theoretical support and motivation for our research. In our study, MIF-rich exosomes could promote NPC by inhibiting the ferroptosis of macrophages when accompanied by RSL3. However, since we found MIF could also cause slight fluctuations in the values of ROS and CCK8 in macrophages, the existence of other cell death methods could be further explored.

Exosomes can transport biologically active molecules such as proteins, mRNA, DNA, and noncoding RNA from donor cells to recipient cells and cause reprogramming of target cells and changes in epigenetic factors (Abak et al., 2018). Our research focused on the transfer of exosomal proteins derived from NPC cells to macrophages, which later played a role in macrophages in the development of NPC. It has been reported that MIF is abnormally overexpressed in human HNSC, which promotes the cancer progression (Li et al., 2004). Costa-Silva et al. found that pancreatic ductal adenocarcinomas (PDAC)-derived exosomal MIF induced bone marrow-derived cell migration to the liver and macrophage ablation blocked the pro-metastatic effect of PDAC-derived exosomes in the liver (Costa-Silva et al., 2015). This prompts us to study whether MIF in exosomes can also affect the function of macrophages in NPC. Notably, the serum samples of NPC patients showed significantly higher exosomal MIF levels than normal control serum samples, which might aid in the development of liquid biopsy technology in the future.

GPX4 is a unique enzyme that protects cells against membrane lipid peroxidation and maintains redox homeostasis, by reducing highly reactive lipid hydroperoxides (LOOH) to non-reactive lipid alcohols (Dixon et al., 2012b; Yang et al., 2014). GPX4 is the key upstream regulator of ferroptosis, a form of regulated cell death characterized by the iron-dependent accumulation of LOOH to lethal levels (Seibt et al., 2019). We checked the literature and found that the cGAS–STING pathway might be involved (Jia et al., 2020). Ferroptosis is an iron-dependent non-apoptotic cell death that can be elicited by pharmacological inhibiting the cystine/glutamate antiporter, system Xc^−^(type I) or directly binding and loss of activity of GPx4 (Type II) in cancer cells with high level RAS-RAF-MEK pathway activity or p53 expression, but not in normal cells (Imai et al., 2017).

Due to extensive genomic changes, the therapeutic effect for tumor cells is limited, and recent research has focused on achieving an in-depth understanding and utilization of the microenvironment (Affo et al., 2017). The life and biology of professional phagocytes (macrophages, microglia) are related to dangerous pro-oxidative environments (Brüne et al., 2013). However, it is well known that phagocytes are not sensitive to ferroptosis (Conrad et al., 2018). Macrophages are vital phagocytic cells in the immune microenvironment. Their survival is closely related to the occurrence, development, and even metastasis of tumors. Our research shows that the accumulation of MIF in macrophages protects these cells from ferroptosis to facilitate the malignant biological behavior of tumors. However, the pathways specifically affected by MIF in macrophages and their effects on ferroptosis, as well as how MIF was delivered to and contained by macrophages through exosomes, remained to be explored.

## Data Availability

Publicly available datasets were analyzed in this study. This data can be found here: https://portal.gdc.cancer.gov/projects/TCGA-HNSC, accession number phs000178.v11.p8.
