# Supplementary material for: Exosomal MIF Derived From Nasopharyngeal Carcinoma Promotes Metastasis by Repressing Ferroptosis of Macrophages
Source: Front Cell Dev Biol. 2021 Dec 31;9:791187. doi: 10.3389/fcell.2021.791187 (PMC8758577; doi:10.3389/fcell.2021.791187)

**Table S1. Clinical information of patients in the NPC study, related to Figure 1,2 and Table 1.**

| No. | Year of diagnosis | Age | Gender (M: Male; F: Female) | TNM clinical stage | Survival status (Dead: 0; Alive: 1) | MIF score |
| --- | --- | --- | --- | --- | --- | --- |
| 1 | 2006/3/9 | 63 | M | T1N0M0 | 0 | 2 |
| 2 | 2008/6/6 | 60 | M | T1N0M0 | 1 | 1 |
| 3 | 2008/7/3 | 59 | M | T1N0M0 | 1 | 1 |
| 4 | 2005/12/27 | 53 | M | T2N0M0 | 0 | 6 |
| 5 | 2006/1/18 | 56 | M | T2N0M0 | 1 | 4 |
| 6 | 2006/5/8 | 45 | M | T2N1M0 | 0 | 6 |
| 7 | 2006/11/24 | 63 | M | T2N0M0 | 1 | 2 |
| 8 | 2007/4/17 | 44 | M | T2N1M0 | 0 | 9 |
| 9 | 2007/4/27 | 59 | M | T1N1M0 | 0 | 6 |
| 10 | 2007/8/8 | 56 | M | T1N1M0 | 0 | 6 |
| 11 | 2007/11/28 | 45 | M | T2N0M0 | 0 | 2 |
| 12 | 2008/1/17 | 63 | M | T2aN1M0 | 1 | 6 |
| 13 | 2008/6/21 | 56 | M | T2N1M0 | 1 | 4 |
| 14 | 2010/1/23 | 31 | F | T1N2Mx | 1 | 6 |
| 15 | 2009/4/2 | 64 | M | T1N1M0 | 1 | 6 |
| 16 | 2009/6/18 | 62 | M | T1N1M0 | 0 | 6 |
| 17 | 2009/12/11 | 41 | M | T2N1M0 | 1 | 2 |
| 18 | 2009/11/16 | 62 | F | T1N1M0 | 0 | 6 |
| 19 | 2010/2/2 | 43 | F | T2N0M0 | 1 | 4 |
| 20 | 2010/4/21 | 44 | M | T2N0M0 | 1 | 3 |
| 21 | 2010/5/5 | 64 | F | T1N1M0 | 1 | 3 |
| 22 | 2010/6/8 | 62 | F | T2N1M0 | 1 | 4 |
| 23 | 2010/6/14 | 45 | M | T1N1M0 | 0 | 8 |
| 24 | 2010/6/15 | 77 | F | T1N1M0 | 1 | 2 |
| 25 | 2010/6/28 | 46 | F | T1N1M0 | 1 | 4 |
| 26 | 2010/8/30 | 70 | F | T1N1M0 | 1 | 4 |
| 27 | 2010/12/17 | 60 | M | T1N1M0 | 0 | 9 |
| 28 | 2005/8/3 | 68 | M | T3N0M0 | 0 | 6 |
| 29 | 2005/8/15 | 54 | M | T3N0M0 | 1 | 9 |
| 30 | 2005/10/10 | 57 | M | T3N2M0 | 0 | 12 |
| 31 | 2006/9/1 | 46 | F | T4N1M0 | 1 | 12 |
| 32 | 2006/12/14 | 54 | M | T2N2M0 | 1 | 12 |
| 33 | 2007/1/29 | 57 | M | T3N2M0 | 1 | 9 |
| 34 | 2006/12/29 | 62 | M | T2N2M0 | 0 | 8 |
| 35 | 2006/11/17 | 57 | M | T2N2M0 | 0 | 9 |
| 36 | 2007/5/11 | 65 | M | T2N2M0 | 0 | 12 |
| 37 | 2007/7/31 | 54 | M | T3N1M0 | 0 | 6 |
| 38 | 2007/8/7 | 61 | M | T3N1M0 | 1 | 6 |
| 39 | 2007/8/24 | 69 | F | T1N2M0 | 1 | 9 |
| 40 | 2007/8/22 | 49 | M | T3N1M0 | 0 | 9 |
| 41 | 2007/10/29 | 33 | M | T1N2M0 | 1 | 3 |
| 42 | 2007/11/15 | 59 | F | T1N2M0 | 1 | 12 |
| 43 | 2007/9/25 | 52 | F | T2N2M0 | 0 | 9 |
| 44 | 2007/11/19 | 45 | M | T2N2M0 | 1 | 8 |
| 45 | 2007/9/18 | 49 | M | T1N2M0 | 1 | 8 |
| 46 | 2008/3/14 | 50 | M | T3N2M1 | 0 | 16 |
| 47 | 2007/12/21 | 62 | M | T3N1M0 | 0 | 12 |
| 48 | 2007/12/27 | 66 | M | T3N1M0 | 1 | 8 |
| 49 | 2008/8/5 | 74 | M | T2N2M0 | 0 | 12 |
| 50 | 2008/6/3 | 42 | M | T3N2M0 | 0 | 12 |
| 51 | 2008/8/13 | 57 | F | T3N2M0 | 1 | 12 |
| 52 | 2009/4/21 | 53 | M | T2bN2M0 | 1 | 9 |
| 53 | 2009/4/24 | 58 | F | T2aN2M0 | 1 | 6 |
| 54 | 2009/5/14 | 44 | M | T2N2M0 | 0 | 12 |
| 55 | 2009/7/14 | 53 | M | T1N2M0 | 0 | 12 |
| 56 | 2009/9/25 | 43 | M | T3N1M0 | 1 | 8 |
| 57 | 2009/10/29 | 70 | M | T3N1M0 | 0 | 12 |
| 58 | 2010/4/28 | 63 | M | T3N0M0 | 1 | 4 |
| 59 | 2010/6/8 | 68 | F | T3N2M0 | 1 | 12 |
| 60 | 2010/6/14 | 48 | F | T1N2M0 | 1 | 12 |
| 61 | 2010/7/21 | 48 | F | T2N2M0 | 1 | 9 |
| 62 | 2005/11/4 | 42 | F | T4N0M0 | 1 | 9 |
| 63 | 2005/12/12 | 50 | F | T3N3M0 | 0 | 12 |
| 64 | 2006/8/9 | 48 | F | T2N3M0 | 1 | 9 |
| 65 | 2006/9/1 | 63 | M | T4N1M0 | 0 | 12 |
| 66 | 2006/12/29 | 60 | M | T2N1M1 | 0 | 12 |
| 67 | 2007/5/22 | 27 | F | T4N1M0 | 0 | 12 |
| 68 | 2007/6/25 | 54 | M | T2N3M0 | 1 | 12 |
| 69 | 2007/8/8 | 67 | M | T4N1M0 | 0 | 12 |
| 70 | 2007/6/29 | 53 | M | T1N2M1 | 0 | 12 |
| 71 | 2007/11/28 | 60 | F | T4N2M0 | 1 | 16 |
| 72 | 2008/2/26 | 49 | F | T4N1M0 | 1 | 12 |
| 73 | 2008/4/18 | 76 | M | T4N2M0 | 0 | 12 |
| 74 | 2008/12/2 | 40 | M | T4N0M0 | 1 | 12 |
| 75 | 2008/8/12 | 51 | M | T4N0M0 | 1 | 9 |
| 76 | 2010/4/26 | 66 | M | T4N1M0 | 0 | 12 |
| 77 | 2010/8/13 | 55 | M | T4N3M0 | 0 | 16 |
| 78 | 2008/3/26 | 43 | M | T1N1M0 | 1 | 3 |
| 79 | 2008/7/18 | 43 | F | T1N1M0 | 0 | 9 |
| 80 | 20094167 | 36 | M | T1N1M0 | 1 | 2 |

**CNE2 transfection efficiency of knockdown MIF lentivirus and knockdown efficiency of MIF in exosomes**


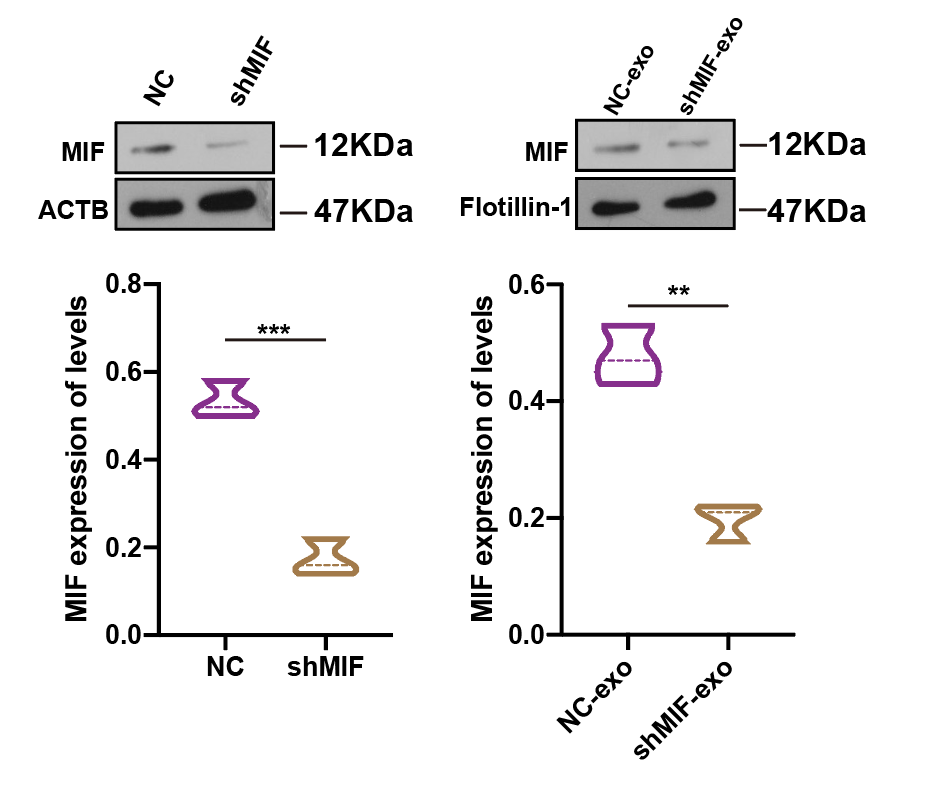

Supplement: Supplementary file 1 [file Table1.DOCX]
